# Microplastic Occurrence and Polymer Confirmation in Five Houston Urban Waterways

**DOI:** 10.1007/s00128-026-04326-8

**Published:** 2026-09-15

**Authors:** Yasemin Umurhan, Tej B. Limbu, Tuan Phan

**Affiliations:** 1 Department of Chemistry, Texas Southern University, Houston, TX USA; 2 Department of Physical and Applied Sciences, University of Houston-Clear Lake, Houston, TX USA

**Keywords:** Microplastics, Freshwater, Houston, Raman spectroscopy, ATR-FTIR, Polymer confirmation

## Abstract

Polymer-confirmed freshwater microplastic data remain limited for Houston urban waterways. We screened five waterways during one winter baseflow period using inverted microscopy with Raman and ATR-FTIR confirmation of particles from 75 μm to 2 mm. Visually identified suspected plastic particles were observed in samples from all five waterways, with concentrations ranging from 8.7 to 9.2 particles L⁻¹. Fragments dominated most samples, while Buffalo Bayou and Vince Bayou showed higher fiber contributions. Spectroscopic analysis screened for a broad range of synthetic polymers and confirmed the occurrence of polyethylene terephthalate (PET), polyethylene (PE), and polypropylene (PP) among the analyzed particles. Because each waterway was represented by one composite sample and limited blank controls, the results should be interpreted as an initial descriptive screening dataset rather than as statistically generalizable site comparisons or quantitative polymer-abundance measurements.

**Supplementary Information:**

The online version contains supplementary material available at https://doi.org/10.1007/s00128-026-04326-8.

## Introduction

Urban rivers and bayous receive microplastics through multiple pathways, including stormwater runoff, wastewater-linked inputs, atmospheric deposition, roadway-associated particles, and diffuse litter (Liu et al. 2023; O’Brien et al. 2023; Sun et al. 2022; Troost et al. 2024). Houston’s bayou network drains a large metropolitan landscape before connecting with downstream coastal waters, but localized freshwater data with polymer confirmation remain limited for upstream urban waterways.

Visual microscopy is useful for rapid enumeration, but natural fibers and other nonplastic debris can be misclassified without confirmatory analysis (Araujo et al. 2018; Ribeiro-Claro et al. 2017; Umurhan et al. 2025). This study provides a concise screening baseline for five Houston waterways sampled during one winter baseflow period. The objective was to document the occurrence, size fractions, morphologies, and colors of visually identified suspected plastic particles and to confirm the presence of selected polymer types using Raman and ATR-FTIR spectroscopy. The study was designed as an initial descriptive screening assessment and not as a quantitative polymer-abundance study or a formal statistical comparison among waterways.

## Materials and Methods

### Study Design and Sample Collection

Five surface-water sites were selected within the Houston metropolitan area: Brays Bayou, Buffalo Bayou, Greens Bayou, the San Jacinto River, and Vince Bayou (Table 1). The sites were selected to provide geographic coverage of major waterways draining urbanized portions of metropolitan Houston and to include waterways influenced by residential, commercial, transportation, and industrial land uses. Site selection also considered safe public access and the feasibility of collecting the required sample volume under comparable winter baseflow conditions. Sampling was conducted in January-February 2025 under winter baseflow conditions to reduce the immediate influence of storm-driven runoff. At each site, fifteen 1-L grab samples were collected in pre-rinsed glass Mason jars and combined into one 15-L composite. Samples were transported on ice and stored at 4 °C before processing.

Powder material containing multiple particles recovered from each sample and size fraction was subjected to spectroscopic analysis. Raman spectroscopy was used preferentially when the powder material could be positioned within the laser sampling area and produced an interpretable Raman signal. ATR-FTIR was used as a complementary technique when sufficient material was available to establish effective contact with the ATR crystal or when Raman analysis was limited by fluorescence, weak signal intensity, or sample heating. Where material availability permitted, both techniques were applied to provide complementary polymer confirmation. Because the analyzed powder samples contained multiple particles rather than individually isolated particles, a representative spectrum may contain diagnostic features of more than one polymer when different polymer particles are present within the analyzed area. Detection of multiple polymer signatures in a spectrum therefore does not indicate that a single particle was identified as more than one polymer. The spectroscopic results for each site and size fraction are provided in the Supplementary Material.

### Sample Preparation and Microscopy

Sample processing followed a rapid oxidative digestion and sieve-based workflow adapted from freshwater microplastic methods (Nayebi et al. 2023; Sharma et al. 2024). Organic matter was reduced by adding 30% H₂O₂ and allowing digestion for 24 h at room temperature without deliberate heating. Samples were sequentially passed through stainless-steel sieves of 2 mm, 180 μm, and 75 μm. No suspected plastic particles larger than 2 mm were observed; therefore, the 180 μm to 2 mm and 75 to 180 μm fractions were retained. Material retained on each stainless-steel sieve was rinsed with deionized water and maintained as a separate size fraction. After rinsing, each sieve was covered with aluminum foil and allowed to dry at room temperature for at least 24 h. The dried retained material was transferred using clean metal or glass tools into separately labeled glass containers, which were sealed with aluminum foil until analysis. The retained material was then examined using a Motic AE2000 inverted microscope at 40× magnification. During microscopic screening, particles were classified as suspected plastic particles when they lacked visible cellular or organic structures, fibers exhibited relatively uniform thickness along their length, and particles showed comparatively homogeneous color and morphology. These visual criteria were used for preliminary classification only and were not considered sufficient for polymer confirmation.

### Spectroscopic Confirmation, QA/QC, and Data Treatment

Samples analyzed by Raman spectroscopy and/or ATR-FTIR consisted of powder material containing multiple particles from each site and size fraction rather than individually isolated particles. Therefore, a spectrum may contain diagnostic features of more than one polymer when different polymer particles are present within the analyzed area. Detection of multiple polymer signatures in a spectrum does not indicate that a single particle was identified as more than one polymer. Raman spectra were acquired using a Thermo Scientific DXR2 SmartRaman spectrometer equipped with a 785-nm excitation laser and a 400 lines mm^−1^ diffraction grating. The laser power was 10 mW. Each sample spectrum was collected using an integration time of 1 s per scan and 20 accumulations, while the corresponding background spectrum was collected using an integration time of 1 s and 16 accumulations. The spectral resolution ranged from approximately 2.4 to 4.3 cm^−1^. The SmartRaman configuration used in this study employed a fiber-optic sampling probe and did not use a microscope objective. The approximate laser spot diameter at the sample was 3.1 mm. ATR-FTIR spectra were collected using an Agilent Cary 630 instrument over 4000–400 cm^−1^.

Raman and ATR-FTIR spectra were subjected to baseline correction and normalization before spectral comparison. Spectra were compared with reference spectra available through the Open Specy spectral library, while OMNIC software was used for spectral processing and evaluation. A library match of at least 70% was used as the preliminary criterion for polymer identification. A polymer assignment was accepted only when the library match met or exceeded this threshold and the measured spectrum also displayed characteristic diagnostic bands or peaks consistent with the proposed polymer. Polymer assignments were therefore based on the combined results of library matching and manual evaluation of diagnostic Raman shifts or ATR-FTIR absorption bands. Spectra that did not meet both the library-match criterion and the diagnostic-peak criterion were not assigned to a polymer type. Raman and infrared spectroscopy are widely used for microplastic polymer confirmation when visual identification alone is insufficient (Araujo et al. 2018; Ribeiro-Claro et al. 2017; Umurhan et al. 2025). Representative microscopic images, Raman spectra, ATR-FTIR spectra, and diagnostic polymer-assignment peaks are provided in the Supplementary Material.

Sample collection and laboratory processing were conducted using glass Mason jars, stainless-steel sieves, glass storage containers, aluminum foil covers, and metal or glass handling tools. Plastic laboratory containers and plastic filtration materials were not used during sample collection, sieving, drying, or storage. All glassware, sieves, and handling tools were rinsed with deionized water before use. Retained material was dried and stored while covered to minimize airborne contamination. No suspected plastic particles were observed during microscopic examination of the airborne-exposure blank. However, because the blank was not carried through digestion, sieving, drying, and spectroscopic analysis, it did not constitute a full procedural blank. These limitations are important because laboratory particles can occur in blanks and can affect microplastic interpretation (Gaston et al. 2020; Munno et al. 2023). No inferential statistical comparisons among sites were attempted.

## Results and Discussion

Visually identified suspected plastic particles were observed in samples from all five waterways (Table 1). Total concentrations varied narrowly, from 8.7 particles L^−1^ in Brays Bayou to 9.2 particles L^−1^ in Buffalo Bayou. The narrow range is consistent with a rapid baseflow screening design and should not be interpreted as evidence that the sites are statistically similar or different.

Visually identified fragments dominated the suspected-particle counts in Brays Bayou, Greens Bayou, the San Jacinto River, and Vince Bayou. Buffalo Bayou and Vince Bayou showed the largest fiber contributions, at 3.2 and 2.5 particles L^−1^, respectively. These observations of suspected fibers may help guide later pathway-focused sampling, but the present design cannot distinguish among wastewater-linked, atmospheric, textile-derived, stormwater, or other urban sources. Similar caution is recommended in freshwater microplastic studies because multiple point and nonpoint pathways can overlap within the same watershed (Kurki-Fox et al. 2023; Troost et al. 2024).

**Table 1:** Sampling locations and descriptive results for visually identified suspected plastic particles in five Houston waterways. Concentrations are based on one 15-L composite sample per waterway

Waterway	Coordinates	Fibers, 75–180 μm (particles L^−1^)	Fibers, 180 μm–2 mm (particles L^−1^)	Fragments, 75–180 μm (particles L^−1^)	Fragments, 180 μm–2 mm (particles L^−1^)	Total (particles L^−1^)	Dominant colors
Brays Bayou	29°42′11″N, 95°33′56″W	–	0.6	4.8	3.3	8.7	White, black, light brown
Buffalo Bayou	29°45′52″N, 95°21′55″W	1.5	1.7	3.2	2.8	9.2	White, black
Greens Bayou	29°46′37″N, 95°11′53″W	0.6	0.5	4.2	3.5	8.9	White, black
San Jacinto River	29°49′45.4″N, 95°05′07.4″W	–	0.0	4.7	4.1	8.8	White, black
Vince Bayou	29°42′28″N, 95°12′55″W	1.4	1.3	3.4	3.0	9.1	White, black

Spectroscopic analysis of the analyzed particles identified only PET, PE, and PP; no other polymer types were identified among the particles subjected to spectroscopic analysis (Table 2). PET was observed among the analyzed particles from all five waterways and in eight of the ten site-size-fraction combinations. PE was observed in six site-size-fraction combinations, and PP was observed in four. Because the spectroscopic results were not used to determine the polymer identity of every visually counted particle, the findings are interpreted as evidence of polymer occurrence rather than quantitative polymer-frequency or polymer-abundance data.

**Table 2:** Polymer types observed among the suspected particles subjected to Raman and/or ATR-FTIR analysis in each waterway and size fraction.

Waterway	Size fraction	PET	PP	PE
Brays Bayou	180 μm to 2 mm	Observed	Not observed	Observed
Brays Bayou	75 to 180 μm	Not observed	Not observed	Observed
Buffalo Bayou	180 μm to 2 mm	Observed	Observed	Not observed
Buffalo Bayou	75 to 180 μm	Observed	Observed	Not observed
Greens Bayou	180 μm to 2 mm	Observed	Not observed	Observed
Greens Bayou	75 to 180 μm	Observed	Not observed	Observed
San Jacinto River	180 μm to 2 mm	Observed	Not observed	Observed
San Jacinto River	75 to 180 μm	Observed	Observed	Observed
Vince Bayou	180 μm to 2 mm	Observed	Observed	Not observed
Vince Bayou	75 to 180 μm	Observed	Observed	Not observed

“Observed” indicates that at least one analyzed particle produced an acceptable library match and corresponding diagnostic spectral features for the listed polymer. “Not observed among analyzed particles” indicates that the polymer was not identified among the particles analyzed and does not demonstrate that the polymer was absent from the complete sample

The technical significance of the study is its localized, polymer-confirmed baseline for Houston freshwater systems. The data support the feasibility of combining low-volume composite sampling, microscopy, and targeted Raman/ATR-FTIR confirmation for rapid urban waterway screening. This approach is useful for identifying where more rigorous replicated sampling, event-based monitoring, and expanded blank controls should be prioritized.

The main shortcomings are the single composite sample per waterway, the lack of replicate full procedural blanks, and the representative rather than exhaustive spectroscopic confirmation. Future work should include repeated seasonal and post-rainfall sampling, procedural blanks carried through digestion and sieving, recovery checks, and polymer-specific confirmation of a larger fraction of counted particles. These steps would allow stronger evaluation of temporal variability, site differences, and likely transport pathways in Houston watersheds.

## Supplementary Information

The online version contains supplementary material including representative microscopic images, Raman spectra, ATR-FTIR spectra, and diagnostic polymer-assignment tables.

Supplementary file1 (DOCX 3203 kb)

## Data Availability

The datasets generated and/or analyzed during the current study are available from the corresponding author upon reasonable request.
