# Supplementary material for: Microplastic Occurrence and Polymer Confirmation in Five Houston Urban Waterways
Source: Bull Environ Contam Toxicol. 2026 Sep 15;117(3):54. doi: 10.1007/s00128-026-04326-8 (PMC13578043; doi:10.1007/s00128-026-04326-8)
Supplement: Supplementary file 1 [file 128_2026_4326_MOESM1_ESM.docx]

**Supplementary Material**

**Microplastic Occurrence and Polymer Confirmation in Five Houston Urban Waterways**

Yasemin Umurhan¹, Tej B. Limbu², and Tuan Phan¹*

¹Department of Chemistry, Texas Southern University, Houston, Texas, USA

²Department of Physical and Applied Sciences, University of Houston-Clear Lake, Houston, Texas, USA

*Corresponding author: Tuan Phan, Email: tuan.phan@tsu.edu

**Artwork Software and Instrument Output Statement**

Figures in this supplementary material were generated directly from analytical instrument software. Raman and ATR-FTIR spectra were obtained using the respective instrument data acquisition software and exported as graphical outputs for presentation. Microscopic images were captured using the microscope imaging system under standard illumination and magnification settings. The exported images and spectra were minimally processed for clarity (such as cropping, labeling, and formatting) using Microsoft Excel, Microsoft PowerPoint, or Microsoft Paint. No alterations were made that would modify the original analytical data.

| (a) Brays Bayou | | (b) Buffalo Bayou | | (c) Greens Bayou | |
| --- | --- | --- | --- | --- | --- |
|  | (d) San Jacinto River | | (e) Vince Bayou | |  |

**Figure S1**. Representative microscopic images of MP particles (fragments and fibers) collected from (a) Brays Bayou, (b) Buffalo Bayou, (c) Greens Bayou, (d) the San Jacinto River, and (e) Vince Bayou. Images were acquired under standard illumination at 40× magnification. Scale bar = 100 μm.

**Figure S2**. Representative Raman spectra obtained from powder samples of the 180 µm–2 mm fraction from (a) Brays Bayou, showing diagnostic Raman bands characteristic of PE; (b) Buffalo Bayou, showing diagnostic Raman bands characteristic of PP; (c) Greens Bayou, showing diagnostic Raman bands characteristic of PET and PE; (d) San Jacinto River, showing diagnostic Raman bands characteristic of PET and PE; and (e) Vince Bayou, showing diagnostic Raman bands characteristic of PET. Labeled Raman bands indicate the diagnostic spectral features used for polymer identification. Corresponding band assignments and reference values are provided in Supplementary Table S1.

**Figure S3.** Representative Raman spectra obtained from powder samples of the 75–180 µm fraction from (a) Brays Bayou, showing diagnostic Raman bands characteristic of PE; (b) Buffalo Bayou, showing diagnostic Raman bands characteristic of PET; (c) Greens Bayou, showing diagnostic Raman bands characteristic of PET and PE; (d) San Jacinto River, showing diagnostic Raman bands characteristic of PET and PE; and (e) Vince Bayou, showing diagnostic Raman bands characteristic of PET. Labeled Raman bands indicate the diagnostic spectral features used for polymer identification. Corresponding band assignments and reference values are provided in Supplementary Table S1.

**Figure S4**. Representative ATR-FTIR spectra obtained from powder samples of the 180 µm–2 mm fraction from (a) Brays Bayou, showing diagnostic absorption bands characteristic of PET and PE; (b) Buffalo Bayou, showing diagnostic absorption bands characteristic of PET and PP; (c) Greens Bayou, showing diagnostic absorption bands characteristic of PE; (d) San Jacinto River, showing diagnostic absorption bands characteristic of PE; and (e) Vince Bayou, showing diagnostic absorption bands characteristic of PET and PP. Corresponding band assignments and reference values are provided in Supplementary Table S2.

**Figure S5.** Representative ATR-FTIR spectra obtained from powder samples of the 75–180 µm fraction from (a) Brays Bayou, showing diagnostic absorption bands characteristic of PE; (b) Buffalo Bayou, showing diagnostic absorption bands characteristic of PET and PE; (c) Greens Bayou, showing diagnostic absorption bands characteristic of PET and PE; (d) San Jacinto River, showing diagnostic absorption bands characteristic of PET and PP; and (e) Vince Bayou, showing diagnostic absorption bands characteristic of PET and PP. Corresponding band assignments and reference values are provided in Supplementary Table S2.

**Table S1.** Raman spectral characteristics of MPs identified in each waterway.

| **Waterway** | **Size** | **PET** | **PP** | **PE** |
| --- | --- | --- | --- | --- |
| Brays Bayou | 180 µm – 2 mm | Not detected | Not detected | 2840–2882 cm⁻¹ (C–H stretching),  1436 cm⁻¹ (CH₂ deformation), and  1291 cm⁻¹ (wagging) |
|  | 75 µm – 180 µm | Not detected | Not detected | 2849–2882 cm⁻¹ (C–H stretching),  1457 cm⁻¹ and 1435 cm⁻¹  (CH₂ bending),  1293 cm⁻¹ (CH₂ rocking) |
| Buffalo Bayou | 180 µm – 2 mm | Not detected | 2845–2881 cm⁻¹ (C–H stretch),  1435 cm⁻¹,  1324 cm⁻¹,  1293 cm⁻¹ (CH₂ deformation) | Not detected |
|  | 75 µm – 180 µm | 1724 cm⁻¹ (C=O stretching),  1613 cm⁻¹ (C=C aromatic vibration)  1461 cm⁻¹ (CH₂ bending),  1284–1095 cm⁻¹ (C–C, C–O stretching)  853 cm⁻¹ (aromatic deformation),  628 cm⁻¹ (aromatic deformation) | Not detected | Not detected |
| Greens Bayou | 180 µm – 2 mm | 1725 cm⁻¹ (C=O stretching),  1612 cm⁻¹ (C=C aromatic vibration)  626 cm⁻¹ (ring deformation) | Not detected | 2950–2879 cm⁻¹ (C–H stretching),  1456–1329 cm⁻¹ (CH₂ bending),  1292 cm⁻¹ (CH₂ bending) |
|  | 75 µm – 180 µm | 1721 cm⁻¹ (C=O stretching),  1612 cm⁻¹ (C=C aromatic vibration.)  1123–1057 cm⁻¹  (C–O, ring deformations) | Not detected | 2882–2846 cm⁻¹ (C–H stretching),  1437–1291 cm⁻¹ (CH₂ bending and deformation), |
| San Jacinto River | 180 µm – 2 mm | 1722 cm⁻¹ (C=O stretching),  1610 cm⁻¹ (C=C aromatic vibration.)  804 cm⁻¹ (C–H aromatic ring bending), | Not detected | 2961–2879 cm⁻¹ (C–H stretch),  1454 cm⁻¹ (CH₂ bending),  1326 cm⁻¹ and 1292 cm⁻¹ (C–H deformation) |
|  | 75 µm – 180 µm | 1724 cm⁻¹ (C=O stretching),  1614 cm⁻¹ (C=C aromatic vibration.)  804 cm⁻¹ (C–H aromatic ring bending),  856 cm⁻¹ and 627 cm⁻¹ (aromatic deformation) | Not detected | 2884 cm⁻¹ (C–H stretching), 1454 cm⁻¹ (CH₂ bending),  1291 cm⁻¹ (CH₂ deformation) |
| Vince Bayou | 180 µm – 2 mm | 1724 cm⁻¹ (C=O stretching),  1616 cm⁻¹ (C=C aromatic vibration),  1128 cm⁻¹ (C–O stretching)  1459 cm⁻¹ (CH₂/CH₃ bending),  1294 cm⁻¹ (C–C stretch), 631 cm⁻¹ (aromatic ring bend) | Not detected | Not detected |
|  | 75 µm – 180 µm | 1724 cm⁻¹ (C=O stretch),  1612 cm⁻¹ (C=C aromatic vibration.)  1288 cm⁻¹ (CH₂/CH₃ bending vibration.),  626 cm⁻¹ and 853 cm⁻¹ (ring out-of-plane bending) | Not detected | Not detected |

**Table S2.** ATR-FTIR spectral characteristics of MPs identified in each waterway.

| **Waterway** | **Size** | **PET** | **PP** | **PE** |
| --- | --- | --- | --- | --- |
| Brays Bayou | 180 µm – 2 mm | 2922 cm⁻¹ (C–H stretching)  1715 cm⁻¹ (C=O), 1241–1092 cm⁻¹ (C–O stretching) | Not detected | 2922 cm⁻¹ (C–H stretching)  1461–1409 cm⁻¹ (CH₂/CH₃ bending),  723 cm⁻¹ (C–H rocking) |
|  | 75 µm – 180 µm | Not detected | Not detected | 2915–2848 cm⁻¹ (C–H stretching),  1469 cm⁻¹ (C–H bending), 716 cm⁻¹ (C–H rocking) |
| Buffalo Bayou | 180 µm – 2 mm | 1711 cm⁻¹ (C=O), 1241–1088 cm⁻¹ (C–O stretching)  723 cm⁻¹ (C=C stretching) | 2919–2848 cm⁻¹ (C–H stretching),  1375 cm⁻¹ (C–H stretching),  1469 cm⁻¹ (C–H bending) | Not detected |
|  | 75 µm – 180 µm | 1715 cm⁻¹ (C=O), 1237 cm⁻¹, 1088 cm⁻¹ (C–O–C stretching)  723 cm⁻¹ (C=C stretching) | 2915–2840 cm⁻¹ (C–H stretching),  1375 cm⁻¹ (C–H stretching),  1454 cm⁻¹ (C–H bending) | Not detected |
| Greens Bayou | 180 µm – 2 mm | Not detected | Not detected | 2915–2848 cm⁻¹ (C–H stretching),  1469 cm⁻¹ (C–H bending),  719 cm⁻¹ (C–H rocking) |
|  | 75 µm – 180 µm | 1711 cm⁻¹ (C=O), 1245 cm⁻¹, 1088 cm⁻¹, and 1018 cm⁻¹ (C–O stretching) | Not detected | 2915–2848 cm⁻¹ (C–H stretching),  1454 cm⁻¹ (C–H bending),  719 cm⁻¹ (C–H rocking) |
| San Jacinto River | 180 µm – 2 mm | Not detected | Not detected | 2915–2848 cm⁻¹ (C–H stretch),  1469 cm⁻¹ (CH₂/CH₃ bending vibration),  716 cm⁻¹ (C–H rocking) |
|  | 75 µm – 180 µm | 1718 cm⁻¹ (C=O stretching),  1092 cm⁻¹ (C–O str.)  719 cm⁻¹ (C=C stretching) | 2948, 2915, and 2844 cm⁻¹ (C–H stretching)  1454 cm⁻¹ and 1375 cm⁻¹ (C–H bending) | Not detected |
| Vince Bayou | 180 µm – 2 mm | 1707 cm⁻¹ (C=O), 1241–1096 cm⁻¹ (C–O str.)  723 cm⁻¹ (C=C stretching) | 2915–2848 cm⁻¹ (C–H stretching),  1461 cm⁻¹ and  1375 cm⁻¹ (CH₂/CH₃ bending vibration), | Not detected |
|  | 75 µm – 180 µm | 1715 cm⁻¹ (C=O), 1245–1092 cm⁻¹ (C–O stretching)  693 cmˉ¹ and 719 cmˉ¹ (C=C stretching) | 2952 cm⁻¹ , 2919 cm⁻¹, and 2844 cm⁻¹ (C–H stretching),  1454 cm⁻¹ and  1375 cm⁻¹ (CH₂/CH₃ bending vibration), | Not detected |
